# Detection and Phylogenetic Analysis of Extended-Spectrum β-Lactamase (ESBL)-Genetic Determinants in Gram-Negative Fecal-Microbiota of Wild Birds and Chicken Originated at Trimmu Barrage

**DOI:** 10.3390/antibiotics12091376

**Published:** 2023-08-28

**Authors:** Muhammad Adnan Saeed, Aman Ullah Khan, Syed Ehtisham-ul-Haque, Usman Waheed, Muhammad Fiaz Qamar, Aziz ur Rehman, Amar Nasir, Muhammad Arfan Zaman, Muhammad Kashif, Jean-Paul Gonzalez, Hosny El-Adawy

**Affiliations:** 1 Department of Pathobiology, University of Veterinary and Animal Sciences, Lahore, CVAS Campus, 12-Km Chiniot Road, Jhang 35200, Pakistan; amanullah.khan@uvas.edu.pk (A.U.K.); ehtishamsyed@uvas.edu.pk (S.E.-u.-H.); usman.waheed@uvas.edu.pk (U.W.); fiaz.qamar@uvas.edu.pk (M.F.Q.); aziz.rehman@uvas.edu.pk (A.u.R.); arfan.zaman@uvas.edu.pk (M.A.Z.); 2 Department of Clinical Sciences, University of Veterinary and Animal Sciences, Lahore, CVAS Campus, 12-Km Chiniot Road, Jhang 35200, Pakistan; amar.nasir@uvas.edu.pk (A.N.); muhammad.kashif@uvas.edu.pk (M.K.); 3 Department of Microbiology & Immunology, School of Medicine, Georgetown University, Washington, DC 20057, USA; jean.paul.gonzalez@georgetown.edu; 4 Institute of Bacterial Infections and Zoonoses, Friedrich-Loeffler-Institut, 07743 Jena, Germany; 5 Faculty of Veterinary Medicine, Kafrelsheikh University, Kafr El-Sheikh 35516, Egypt

**Keywords:** ESBL, microbiota, wild birds, aquatic birds, Trimmu Barrage, phylogenetics

## Abstract

Extended-spectrum β-lactamases (ESBL) give rise to resistance against penicillin and cephalosporin antibiotics in multiple bacterial species. The present study was conducted to map genetic determinants and related attributes of ESBL-producing bacteria in three wild aquatic bird species and chickens at the “Trimmu Barrage” in district Jhang, Punjab province, Pakistan. To study the prevalence of ESBL-producing bacteria, a total of 280 representative samples were collected from wild bird species; cattle egrets (*Bubulcus ibis*), little egrets (*Egretta garzetta*) and common teals (*Anas crecca*) as well as from indigenous chickens (*Gallus gallus domesticus*) originating from a local wet market. The isolates were confirmed as ESBL producers using a double disc synergy test (DDST) and bacterial species were identified using API-20E and 20NE strips. A polymerase chain reaction (PCR) was used to detect ESBL genetic determinants and for genus identification via 16S rRNA gene amplification. A phenotypic antimicrobial susceptibility test was performed for ESBL-producing isolates against 12 clinically relevant antibiotics using the Kirby–Bauer disk diffusion susceptibility test. A phylogenetic tree was constructed for the sequence data obtained in this study and comparative sequence data obtained from GenBank. The overall prevalence of ESBL-producing bacteria was 34.64% (97/280). The highest percentage (44.28%; 31/70) of ESBL-producing bacteria was recovered from chickens (*Gallus gallus domesticus*), followed by little egrets (*Egretta garzetta*) (41.43%; 29/70), common teal (*Anas crecca*) (28.57%; 20/70) and cattle egrets (*Bubulcus ibis*) (24.28%; 17/70). Five different ESBL-producing bacteria were identified biochemically and confirmed via 16S rRNA gene sequencing, which included *Escherichia coli* (72; 74.23%), *Enterobacter cloacae* (11; 11.34%), *Klebsiella pneumoniae* (8; 8.25%), *Salmonella enterica* (4; 4.12%) and *Pseudomonas aeruginosa* (2; 2.06%). Based on PCR, the frequency of obtained ESBL genes in 97 isolates was *bla*_CTX-M_ (51.55%), *bla*_TEM_ (20.62%), *bla*_OXA_ (6.18%) and *bla*_SHV_ (2.06%). In addition, gene combinations *bla*_CTX-M_ + *bla*_TEM_, *bla*_TEM_ + *bla*_OXA_ and *bla*_CTX-M_ + *bla*_SHV_ were also detected in 16.49%, 2.06% and 1.03% of isolates, respectively. The ESBL gene variation was significant (*p* = 0.02) in different bacterial species while non-significant in relation to different bird species (*p* = 0.85). Phylogenetic analysis of amino acid sequence data confirmed the existence of CTX-M-15 and TEM betalactamases. The average susceptibility of the antibiotics panel used was lowest for both *Klebsiella pneumoniae* (62.5% ± 24.42) and *Salmonella enterica* (62.5% ± 31.08) as compared to *Enterobacter cloacae* (65.90% ± 21.62), *Pseudomonas aeruginosa* (70.83% ± 33.42) and *Escherichia coli* (73.83% ± 26.19). This study provides insight into the role of aquatic wild birds as reservoirs of ESBL-producing bacteria at Trimmu Barrage, Punjab, Pakistan. Hence, active bio-surveillance and environment preservation actions are necessitated to curb antimicrobial resistance.

## 1. Introduction

Antimicrobial resistance (AMR) has been categorized among the top ten lead threats of public health significance by the World Health Organization (WHO) [1]. AMR-associated global human mortality is expected to rise to the hefty figure of 10 million per annum by 2050 as compared to 0.7 million in 2019 [2]. Antibiotics, mainly cephalosporin and penicillin, are vulnerable to structural degradation through a wide variety of bacteria-origin hydrolyzing enzymes known as extended-spectrum β-lactamase (ESBL) [3]. Hundreds of individual strains of bacteria belonging mainly to several species of Enterobacterales and a few non-Enterobacterales orders are known to produce ESBL enzymes [4]. β-lactamases include a wide variety of hydrolytic enzymes that tend to destabilize the structural integrity of β-lactam antibiotics through degradation of the amide bond of the β-lactam ring [4]. ESBL-producing bacteria are used as bio-surveillance markers to map the emergence and extent of antimicrobial resistance (AMR) under one-health research paradigms [5]. Classically, the ESBL-producing bacteria species were thought to be associated with clinical settings which would transfer mainly via nosocomial, and community-acquired channels [6]. However, the larger picture revealed by recent studies shows a complicated and multi-origin spread of resistant bacteria [7,8]. Trans-border dissemination of resistant bacteria includes wild migratory birds, human traffic, farm animals, arthropod-borne vectors, fomites, water, and food chains, although it is not limited to these factors [7,9,10]. Due to multiple factors, including habitat destruction, annual migration, and the search for feeding sites or breeding needs, wild birds often move into urban or peri-urban areas where they may encounter humans and other animals [11]. Many species of bacteria colonize wild aquatic birds as a part of gut microbiota [12]. In this way, resistant bacteria thrive, are carried away to far-furlong areas and are dispensed to new geographical locations and environments as soon as the birds migrate seasonally or interact with other species [13,14]. To feed and breed, the aquatic birds often contact stagnant water sources, streams, garbage landfills, farmed animals, other bird species and even human settlements [13]. For bacteria, AMR is a key component for adaptation in a changing environment under evolutionary process [15]. Two of the main driving forces behind AMR include first the mutations (intrinsic factors), which in most cases decreases the permeability of antibiotics to enter inside the bacterial cells, and second the acquired genetic elements of resistance (e.g., plasmids, integrons, transposons) which are transmissible horizontally [15].

In the Jhang district, Punjab province, Pakistan, Trimmu Barrage controls water flow at the downstream site of the junction between the Chenab and Jhelum rivers. The Trimmu Barrage represents a typical wetland that provides a balanced ecosystem consisting of both terrestrial and aquatic environments. As many as eighty-nine different avian species, including year-round inhabitants and seasonal migratory birds, were recorded at Trimmu Barrage in a previous study [16]. The present study aimed to determine the nature of ESBL genetic determinants in different species of ESBL-producing bacteria isolated from three different wild bird species, including the cattle egret (*Bubulcus ibis*), little egret (*Egretta garzetta*), and common teal (*Anas crecca*) along with indigenous chickens (*Gallus gallus domesticus*) sourced from the local wet-market, by using molecular characterization and phylogenetic analysis. This project was carried out to provide insight into the potential of wild birds to harbor resistant bacteria.

## 2. Results

### 2.1. Prevalence of ESBL-Producing Bacteria and Genetic Determinants in Bird Species

Out of 280 collected samples, 97 (34.6%) were found to contain ESBL-producing bacteria based on the double disc synergy test (DDST) (Table 1). The highest prevalence of ESBL-producing bacteria was recorded in chickens (44.3%). However, among the three studied wild bird species, ESBL-producing bacteria were found to be highest in the little egrets (41.4%), followed by the common teals (28.6%) and cattle egrets (24.3%) (Table 1). PCR detected all ESBL genetic determinants, including *bla*_CTX-M_ (593 bp), *bla*_TEM_ (445 bp), *bla*_SHV_ (1016 bp) and *bla*_OXA_ (296 bp) with varying percentages and combinations in different samples (Table 1). Only *bla*_CTX-M_ and *bla*_TEM_ were detected consistently in all species of birds, while *bla*_SHV_ was detected only in cattle egrets (5.9%) and little egrets (3.5%). Similarly, *bla*_OXA_ was found only in little egrets (13.8%) and chickens (6.5%) (Table 1).

### 2.2. Diversity of ESBL-Producing Bacteria

Five different bacteria species of ESBL producers were identified using biochemical commercial kits, API 20E, and 20NE, (bioMérieux, Craponne, France). Ninety-seven isolates were recovered, including *Escherichia coli* (72; 74.2%), *Enterobacter cloacae* (11; 11.3%), *Klebsiella pneumoniae* (8; 8.3%), *Salmonella enterica* (4; 4.1%) and *Pseudomonas aeruginosa* (2; 2.1%). The distribution of the isolated ESBL-producing bacteria concerning bird species was demonstrated in Table 2. Selected isolates belonging to each species were further confirmed by 16S rRNA gene amplification and sequencing. The sequence data was submitted to the GenBank as *Enterobacter cloacae* (OP744530), *Klebsiella pneumoniae* (OP744528, OP744534), *Salmonella enterica* (OP744581), and *Pseudomonas aeruginosa* (OP745421).

### 2.3. Genetic Determinants Detected in Different ESBL-Producing Bacteria

The One-way ANOVA was used to determine the statistical significance of the ESBL gene variation (types and percentage distribution) in relation to different bird species and bacterial species. The ESBL gene variation was significant (*p* = 0.02) in different bacterial species while non-significant in relation to different bird species (*p* = 0.85). *bla*_CTX-M_ remained the most frequent yet least variable determinant, with a prevalence ranging from 37.5% to 54.5%. *bla*_TEM_ was detected in all species except *K. pneumoniae*. *bla*_SHV_ was rare and appeared in just 2.8% of *E. coli*. Only one *E. cloacae* (9.1%) and five *E. coli* (6.94%) harbored the *bla*_OXA_ gene. A total of three paired ESBL gene combinations were detected; however, *bla*_CTX-M_ and *bla*_SHV_ combinations were found exclusive to *K. pneumoniae* (Table 3).

### 2.4. Phylogenetic Analysis

**Figure 1:**
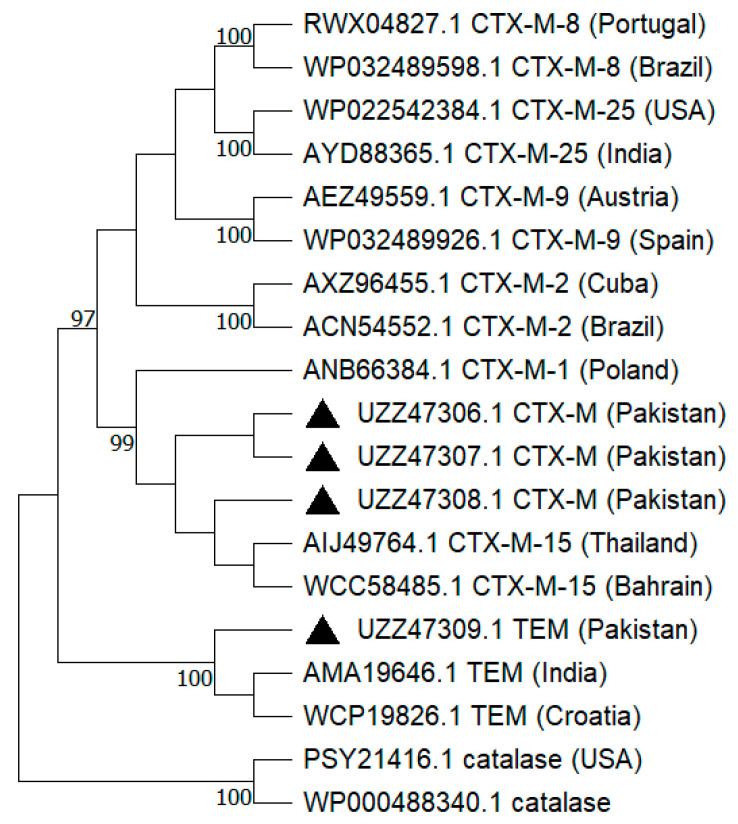
Phylogenetic relationship of amino acid sequence data where present study sequences has been marked with black triangles. The phylogenetic tree was constructed using the maximum likelihood method and JTT matrix-based model. Numeric values below the nodes represent the bootstrap frequency obtained via 1000 replicates.

The phylogenetic tree constructed showed three clades and included an outgroup (catalase enzyme). CTX-M partial protein sequences obtained in this study (UZZ47306.1, UZZ47307.1, and UZZ47308.1) were clustered in clade I and consisted of different groups of CTX-M enzyme. Out of the five groups of CTX-M enzymes (CTX-M-1, CTX-M-2, CTX-M-8, CTX-M-9, and CTX-M-25), the present study sequences were grouped next to the CTX-M-1 and CTX-M-15 (a subtype of CTX-M-1 group). TEM enzyme sequence (UZZ47309.1) appeared in clade II, consisting of TEM sequences from India and Croatia (Figure 1).

### 2.5. Antibiotic Susceptibility Profile

A complete antibiogram of the ESBL-producing bacteria isolated in this study against 12 antimicrobial agents is given in Table 4. All isolated ESBL-producing bacteria in this study were highly resistant to ceftiofur and ampicillin. The resistance of *S. enterica*, *K. pneumoniae*, and *E. cloacae* against tylosin was 50%, 50% and 45.5%, respectively, while *E. coli* and *P. aeruginosa* were sensitive (Table 4). All isolated ESBL-producing bacteria were highly sensitive to the other antimicrobial agents tested in this study.

The overall susceptibility to the panel of antibiotics was lowest for both *K. pneumoniae* (62.5% ± 24.42) and *S. enterica* (62.5% ± 31.08) as compared to *E. cloacae* (65.9% ± 21.62), *P. aeruginosa* (70.8% ± 33.42) and *E. coli* (73.8% ± 26.19) (Table 5). The mean susceptibility of ESBL-producing bacteria was found to be highest for imipenem (96.7%) followed by norfloxacin (87.2%), florfenicol (86.5%), gentamicin (77.7%), colistin (77.5%), enrofloxacin (76.5%), streptomycin (72.3%), neomycin (67.3%), doxycycline (56.5%), tylosin (51%), ceftiofur (38.8%) and ampicillin (17.3%).

## 3. Discussion

Antimicrobial resistance (AMR) is a phenomenon wherein microbes attain the ability to survive a particular concentration of a given antimicrobial substance where they would otherwise be inhibited or killed [17]. Wild birds are reservoirs of pathogens and bacteria resistant to highly and critically important antimicrobials, i.e., ESBL-producing bacteria and MDR strains. Wild birds can occupy different ecological niches and adapt to many urban, suburban and rural environments, representing true environmental sentinels. Due to their ability to move and through the deposition of droppings, birds can play an important role as vectors in the environmental circulation and spread of zoonotic agents, antimicrobial-resistant bacteria and resistance genes [18]. The bio-surveillance system based on monitoring the frequency rates of ESBL-positive bacteria, especially *E. coli*, has become a primary indicator for tracking AMR globally [19]. This system goes beyond a singular focus by embracing the One Health approach, which considers the interconnectedness of animal, human and environmental health in tackling AMR across these sectors [1,5]. Additionally, it facilitates direct comparisons of AMR prevalence and patterns across various regions worldwide.

In Pakistan, most of the selected antibiotics are used in veterinary prescriptions and food animal production, including in the poultry sector [20]. However, some of the selected antibiotics are exclusively used in human medicine (e.g., imipenem and cefotaxime). The antibiotics panel was finally selected under the One Health approach to represent antibiotics of both human and veterinary importance [21]. The emergence of resistant bacteria in wild birds is attributed to environmental contamination with antibiotic residues. The use of tylosin, specifically, was found in 100% (30/30 flocks) of the studied broiler population in Pakistan [21] and vigorous use of tylosin in broiler production was reported previously [20,21]. Although tylosin is primarily active against Gram-positive bacteria, there are reports of a correlation between exposure to tylosin and a change in susceptibility to other antibacterial drugs and even the emergence of multidrug-resistant strains of enterobacteria [22,23]. Such reports suggest that tylosin susceptibility of resistant strains is variable.

There are reports of a high prevalence of ESBL-producing bacteria from clinical and non-clinical sources (mainly poultry and water bodies) in Pakistan [24,25]. The present study included three targeted and commonly found aquatic wild bird species, including cattle egrets (*Bubulcus ibis*), little egrets (*Egretta garzetta*) and common teals (*Anas crecca*) as well as the commercial chickens (*Gallus gallus domesticus*) originated at the diverse aquatic and land environment of the Trimmu Barrage in the district Jhang, Punjab province, Pakistan. Of 280 samples, 97 (34.6%) contained ESBL-positive bacteria based on DDST. The highest prevalence of ESBL-producing bacteria was recorded in chickens (44.3%). Previously, in Pakistan, various studies have reported the existence of ESBL-positive bacteria in commercial chickens with variable prevalence in different regions. Similar to our findings, a study conducted in three districts of the province of Khyber Pakhunkhwa (KPK) (Peshawar, Kohat, and Nowshera) reported an overall prevalence of 43.5% among samples from chickens (liver, spleen and meat) [26]. A study conducted in the Faisalabad metropolitan area (Punjab, Pakistan) reported a relatively low prevalence of ESBL-positive bacteria in poultry (13.7%) as compared to cattle (31%) and humans (55%) (28). From Pakistan, a meta-analysis study on ESBL-producing bacteria revealed an overall prevalence of 40% [24]. Recent studies in Pakistan have reported a much higher prevalence; in a study conducted in Islamabad, ESBL-positive bacteria in chicken was 62.4% [27] and 82% in KPK [28]. Overall, the prevalence of ESBL-positive bacteria in commercial chicken is quite high in Pakistan, as suggested by the current study and previous studies on this subject. This situation is partly explained due to the practice of high antimicrobial use (AMU) for treating bacterial infections, prophylaxis and growth promotion [21]. In Pakistan, the usage of antimicrobials in commercial broiler farming is very high; 22 antibiotics belonging to different classes have been reported to be used on poultry farms, 60% of which have been categorized as critically important antimicrobial classes (CIA) for human medicine by the World Health Organization [20]. Antimicrobials are known to induce selective pressure among normal microflora as well as the avian pathogenic species for evolution into the resistance types [9].

Beta-lactamases (*bla*) are the largest class of antibiotic-resistance genes, which cause resistance to beta-lactam antibiotics (Penicillins and cephalosporins). ESBL enzymes TEM and SHV are known penicillinases. Both have been detected in the present study. According to Beta-Lactamase DataBase (BLDB), 7996 types of beta-lactamases have been identified (http://www.bldb.eu/ accessed on 16 August 2023) [29]. The present study was limited to extended-spectrum beta-lactamases (ESBL) reporting. Amoxicillin was used in the present study as a co-amoxiclav disc while performing a double disc synergy test (DDST). Therefore, ampicillin was used for antibiogram data. Ampicillin was mainly included as a representative of a medically important antibacterial drug; however, it is also used in broiler production in Pakistan. A recent study reported that 91.7% of *Salmonella* spp. recovered from chicken meat in Pakistan were found resistant to ampicillin [30]. A comparable prevalence of 38.18% ESBL-producing *E. coli* has been reported in Bangladesh in migratory wild bird species where various genetic determinants related to ESBL production mechanisms were reported as *bla*_TEM_, *bla*_CMY_, *bla*_CTX-M_ and *bla*_SHV_ were detected in 95.2%, 90.5%, 85.7% and 42.9% of isolates, respectively [31]. In this study, ESBL-producing bacteria were highly prevalent in the little egret (*Egretta garzetta*) (41.4%), followed by the common teal (*Anas crecca*) (28.57%) and cattle egret (*Bubulcus ibis*) (24.28%) at the Trimmu Barrage, Jhang, Punjab, Pakistan. Previous studies conducted in Africa, reported very high prevalence (92.9%) of ESBL-producing bacteria in cattle egrets (*Bubulcus ibis*) as compared to our findings (24.3%), however, consistent to our findings the *bla*_CTX-M-15_ (83.3%) was reported as the major resistance determinant gene along with *bla*_CTX-M-9_ (11.8%) and carbapenemase resistance genes (*bla*_KPC-2_ and *bla*_KPC-3_) [32,33].

To our knowledge, no study has been conducted to report the prevalence of ESBL-producing bacteria originating from the little egret (*Egretta garzetta*) in Pakistan. The present study spotlights the emergence of ESBL *E. coli* in little egrets as the highest (41.43%) among all the studied bird species. A study conducted in Greece reported ESBL genes in 11 out of 12 *E. coli* isolates in fecal samples of different wild and feral bird species (n = 362) but did not find any positive samples from little egrets [34]. This might be attributed to the lower sample size, consisting of only eight samples. A study conducted in China in little egrets reported the ESBL gene prevalence as 46.7% (7/15), with ESBL genes *bla*_OXY_ and *bla*_TEM_ as dominant types [35]. In the present study, a comparable prevalence of ESBL bacteria (41.43%) has been noted in little egrets; however, with a different set of genetic determinants, including *bla*_CTX-M_, *bla*_OXA_, *bla*_SHV_ and *bla*_TEM_. The little egret is considered a successful aquatic bird species colonizing and thriving in wetland environments in the Punjab and Sindh provinces of Pakistan. Sewage, as well as untreated industrial and municipal waste, causes pollution of the wetlands in Pakistan [36]. Heavy metals have been previously detected in the eggshells of little egrets at the Trimmu Barrage area [37]. Given that, it is evident that the environmental contamination of wetlands impacts the types of gut microbiota colonizing the aquatic bird species, especially the little egret. This exposure leads to the colonization of antimicrobial-resistant bacteria in aquatic wildlife [37].

The present study detected 28.6% ESBL *E. coli* in common teals (*Anas crecca*). Previous studies indicated a variable prevalence of ESBL-producing bacteria in ducks: 0% in mallards (*Anas platyrhynchos*) in the Czech Republic [38] and 33.3% (1/3) in mallards in Poland [39]; 31% ESBL *E. coli* were reported from multiple migratory bird species, including common teals (*Anas crecca*) in Pakistan with *bla*_TEM_ dominant gene type [40] and 47% in ducks in Sweden with *bla*_CTX-M-15_ as the dominant ARG [41]. In 2017, the first preliminary report was published that provided evidence of the existence of ESBL-producing *Klebsiella pneumoniae* in wild migratory birds in wetlands in Pakistan [42].

Similar to these findings, the present study reported CTX-M-1 as the predominant group with 88.8% of *bla*_CTX-M_-producing isolates, whereas *bla*_CTX-M-15_ remained the most dominant genotype as per DNA sequencing analysis in Eurasian coot (*Fulica atra*), rosy starling (*Pastor roseus*) and northern shoveler (*Anas clypeata*) [42]. However, our study also reported the existence of combinations of genetic determinants related to ESBL genes (*bla*_CTX-M_ and *bla*_TEM_; 37%), (*bla*_CTX-M_ and *bla*_SHV_; 12.5%) and (*bla*_TEM_ and *bla*_OXA_; 12.5%) in *Klebsiella pneumoniae* isolates in different species of wild birds. The “CTX-M pandemic” is the term used to describe the global spread of ESBL-containing isolates. Since the early 2000s, the CTX-M-type enzymes have been recognized as the most common ESBL category, deposing TEM and SHV as the leading ESBL enzyme types [43]. Previously, CTX-M enzyme genetic determinants have been largely reported in clinical settings, communities, food products, pet animals, environments and farm animals [44]. However, our study reports the existence and dissemination of *bla*_CTX-M_ genes in the fecal microbiota of wild birds and domestic poultry. The *bla*_CTX-M-15_ type gene was identified in the present study, as demonstrated by phylogenetic analysis.

A previous study reported an overall prevalence of ESBL-producing *E. coli* at 17.3% in fecal samples of wild migratory birds in Pakistan [45]. Of these, 88.4% of isolates exhibited multidrug resistance (MDR) characteristics and were found resistant to antibiotics, including cefotaxime, ceftazidime, ampicillin, doxycycline, tetracycline and sulfamethoxazole/trimethoprim, whereas *bla*_CTX-M-15_ was found the most common ESBL gene [45]. Similar to these findings, the ESBL-producing bacteria isolated in the present study were found resistant to multiple antibiotics, and the mean resistance percentage obtained as ampicillin (74.1%), ceftiofur (58.7%), tylosin (32.1%), streptomycin (27.6%), doxycycline (26.8%), colistin (19.8%), enrofloxacin (19.6%), neomycin (17%), gentamicin (15.8%), florfenicol (12.4%), norfloxacin (12.3%) and imipenem (3.3%). These findings indicate that the multidrug resistance phenomenon is common in ESBL-producing bacteria.

Five different species of ESBL-producing bacteria have been identified in our study, including *E. coli*, 74.2%, *E. cloacae*, 11.3%, *K. pneumoniae*, 8.3%, *S. enterica*, 4.1% and *P. aeruginosa*, 2.1%. High prevalence and diversity of ESBL-producing bacteria were noted in Catalonia, including ESBL prevalence (11.5%) in wild birds with *bla*_CMY-2_ (50%) and *bla*_CTX-M-15_ (18%) and various species of bacteria, including *K. pneumoniae* (20%), *C. freundii* (15%), *E. cloacae* (5%), *P. mirabilis* (5%), *Providencia* spp. (5%) and *Serratia marcescens* (2.5%) were also isolated [46]. The nature and diversity of gut microflora in wild birds are dependent on multiple intrinsic (genetic makeup, age, sex, breed, etc.) and extrinsic factors (diet, environment, behavior, social contact, etc.) [47]. Although different species of ESBL-producing bacteria can be isolated by using modified (antibiotics-supplemented) selective culture media, mostly the members of the family *Enterobacteriaceae* are resistant to β-lactam antibiotics. The prevalence of ESBL-producing *E. coli* (74.2%) was notably higher than other co-species of bacteria in the present study. Nevertheless, this finding is inconsistent and varies in different geographical regions and wild bird species. However, it can partially be explained by the remarkable ability of *E. coli* to sustain a wide range of regulatory and metabolic pathways that allow it to survive in different habitats and the alimentary canals of a diverse range of host species [48].

As employed in the present study, 16S rRNA gene sequencing provides an effective tool for bio-surveillance of antimicrobial resistance in wild birds. It can be utilized as a relatively economical, reliable, less labor-intensive and user-friendly alternative to biochemical and serological identification techniques for bacteria, especially in low and middle-income countries. The occurrence of antimicrobial-resistant bacteria in wild bird species could pose a significant challenge for public health in the future. Therefore, it becomes increasingly important to periodically monitor the wild bird species as silent spreaders of resistant bacteria.

## 4. Materials and Methods

### 4.1. Collection of Pooled Fecal Material

The sampling period spanned November 2021 to December 2022. The selection of the three target species was based on relative abundance, convenience sampling and available resources. Selected bird species included two indigenous flying species, cattle egrets (*Bubulcus ibis*) and little egrets (*Egretta garzetta*), along with one winter visitor species, common teals (*Anas crecca*). The birds were observed at their nesting, feeding and breeding hotspots at Trimmu Barrage (district Jhang), Punjab, Pakistan. Sterile aluminum foil sheets (1 m^2^) were spread on the ground at different hotspots to collect fresh droppings. Fresh droppings (3–4) obtained from individual birds of the same species were pooled to prepare a representative sample. Similarly, fresh fecal material was collected from the indigenous chickens (*Gallus gallus domesticus*) originating from a local wet market. A total of 280 representative samples, consisting of seventy samples from each species, were collected in vials of sterile transport media and shipped to the Microbiology laboratory of the College of Veterinary and Animal Sciences, Jhang. Samples were stored at 4 °C until processed further.

### 4.2. Isolation and Confirmation of ESBL-Producing Bacteria

The representative specimens (n = 280) were pre-enriched in 10 mL of tryptone soy broth (CM0129, Oxoid, Hampshire, UK) supplemented with 4 mg/L cefotaxime (Caisson C032-100G, Smithfield, UT, USA) and incubated at 37 °C for 24 h. Pre-enriched broth (100 µL) was streaked on MacConkey agar (Oxoid CM0007, Hampshire, UK) plates supplemented with 4 mg/L cefotaxime and incubated at 37 °C for 24 h [49]. Individual colonies selected from MacConkey agar were sub-cultured in tryptone soy broth to obtain a pure culture. A double disc synergy test (DDST) was used to confirm pure cultures as ESBL producers by following the protocol described in the M100 performance standards for antimicrobial susceptibility testing by the Clinical and Laboratory Standards Institute (CLSI) [50]. Briefly, the 0.5 McFarland standard equivalent test culture was swabbed onto the Mueller-Hinton (CM0337B, Oxoid, Hampshire, UK) agar plates and two antibiotic discs cefotaxime (CTX-30) and amoxicillin/clavulanic acid (AMC-30) were placed at a center-center distance of 20 mm and plates were incubated at 37 °C for 24 h. Expansion of the zone of inhibition of CTX-30 toward the AMC-30 disc was marked as a positive DDST. The species of ESBL bacteria were identified by using API-20E and 20NE strips (bioMérieux, Craponne, France) according to the manufacturer’s instructions.

### 4.3. Molecular Identification of ESBL-Producing Bacteria and Detection of Genetic Determinants of β-Lactamase Associated Genes

The primer sets utilized for the amplification of ESBL genetic determinants (*bla*_CTX-M_, *bla*_TEM_, *bla*_SHV_ and *bla*_OXA_) as well as the 16S rRNA gene sequencing-based genus confirmation of selected isolates have been summarized in Table 6. All the isolates, pre-confirmed as ESBL producers by DDST, were sub-cultured on Tryptone Soy Broth and incubated overnight at 37 °C for 18 h. Fresh broth cultures (1 mL) were processed for extraction of genomic DNA by using WizPrep™ gDNA Kit (Wizbiosolutions, Gyeonggi-do, Republic of Korea) as per manufacturer’s instructions to obtain a DNA concentration of minimally 10 ng/μL. PCR-positive controls for genetic determinants were maintained at the Microbiology laboratory of the College of Veterinary and Animal Sciences, Jhang, Pakistan, through preserved stains, including *E. coli* strain MASMS_A3 (GenBank: ON736876.1) for *bla*_CTX-M_ and *bla*_TEM_ genes. In contrast, *K. pneumoniae* strain MASJG8 (GenBank: OP744534.1) was used for *bla*_SHV_ and *bla*_OXA_ genes. Positive control amplicons were verified through nucleotide sequencing and the BLAST tool of the NCBI. Nuclease-free distilled water was used as a negative control template. Mono-plex PCRs for ESBL genetic determinants were carried out by mixing 25 µL PCR master mix (Dream Taq Green 2x, ThermoFisher, Waltham, MA, USA), template DNA (4 µL), forward and reverse primers (10 pM/µL) as 2 µL per reaction for each primer and completed to total volume 50 µL with nuclease-free water (AM9932, Invitrogen™ Waltham, MA, USA). All PCR tubes were kept in a thermal cycler (Biorad, T100, Hercules, CA, USA) for amplification of the targets under these conditions: pre-denaturation monocycle at 94 °C for 15 min, 30× (denaturation at 94 °C for 30 s, annealing temperatures as per Table 6, for 30 s and extension at 72 °C for 2 min) followed by a 10 min final extension at 72 °C.

The reaction mixture for amplification of 16S rRNA gene was prepared by using 12.5 µL PCR master mix (Dream Taq Green 2x, Thermo Fisher Scientific, Waltham, MA, USA), gDNA (2 µL), primers (fD_1_ and rP_2_) at concentration 10 pM as 1 µL each and completed to total volume 25 µL with nuclease-free water. The thermocycling parameters for amplification of the 16S rRNA gene included initial denaturation at 94 °C for 10 min, 36x (denaturation at 94 °C for 60 s, annealing at 52 °C for 30 s, and extension at 72 °C for 2 min) followed by a single final extension step at 72 °C for 10 min. The PCR products were electrophoresed using the Mupid One Electrophoresis System (NIPPON Genetics, Tokyo, Japan) at 90 volts for 45 min by using agarose gel (1.3%) stained with ethidium bromide at 0.5 μg/mL of the gel. Stained gels were examined with a UV-transilluminator (Fisher Scientific, Hampton, NH, USA) to visualize and capture gel images.

### 4.4. Sequencing of PCR Amplicons and Phylogenetic Analysis

Selected PCR amplicons were tested for DNA concentration (>30 ng/μL) and DNA purity (A_260_/A_280_~1.8) via Nanodrop-1000 Spectrophotometer (ThermoScientific, Greenville, SC, USA) and processed for sanger sequencing by commercial service provider, Beijing Genomics Institute (BGI), Shenzhen (518083), China. For phylogenetic analysis, raw sequence reads were trimmed using BioEdit 7.0 software. Selected sequence data were uploaded to the GenBank database. Sequence data obtained in this study was processed by using the BLAST-p program from the National Center for Biotechnology Information (NCBI) to obtain similar sequence data available at the NCBI. For phylogenetic analysis protein sequence data included present study GenBank accession numbers CTX-M (UZZ47306.1, UZZ47307.1 and UZZ47308.1) and TEM (UZZ47309.1) while comparative sequence data included CTX-M-1 (ANB66384.1), CTX-M-2 (AXZ96455.1, ANB66384.1), CTX-M-8 (RWX04827.1, WP032489598.1), CTX-M-9 (AEZ49559.1, WP032489926.1), CTX-M-15 (AIJ49764.1, WCC58485.1), CTX-M-25 (WP022542384.1, AYD88365.1), TEM (AMA19646.1, WCP19826.1) and catalase as outgroup sequences (PSY21416.1 and WP000488340.1). A phylogenetic tree was constructed using the maximum likelihood method with bootstraps (1000) and the Jones–Taylor–Thornton (JTT) model using MEGA 11 software (64-bit) [55].

### 4.5. Antibiotic Susceptibility Testing

The phenotypic antimicrobial susceptibility test was performed using the Kirby–Bauer disk diffusion susceptibility test for ESBL-producing isolates, including *E. cloacae* (n = 11), *K. pneumoniae* (n = 8), *S. enterica* (n = 4), *P. aeruginosa* (n = 2) and *E. coli* (n = 72) against the panel of 12 antibiotics discs (Oxoid, UK; Condalab, Spain) included streptomycin (10 μg), neomycin (30 µg), gentamicin (10 μg), florfenicol (30 μg), ceftiofur (30 μg), enrofloxacin (5 μg), norfloxacin (10 μg), tylosin (30 µg), ampicillin (10 µg), doxycycline (30 µg), colistin (10 µg) and imipenem (10 μg). Testing and result interpretation protocols were followed as described in the M100s manual of the Clinical Laboratory Standards Institute (CLSI), 2020 [50].

### 4.6. Statistical Analysis

Descriptive statistical analysis, including prevalence, percentages and analysis of variance using One-way ANOVA, was performed using IBM-SPSS (Version-25) software.

## 5. Conclusions

This study described the prevalence, diversity, genotypic characterization, phylogeny and antibiotic susceptibility of the extended-spectrum β-lactamase (ESBL) producing bacteria of wild bird and commercial chicken origin at the Trimmu Barrage (district Jhang), Punjab, Pakistan. The study found that several member species of *Enterobacteriaceae* are ESBL producers, possess a wide range of genetic determinants (*bla*_CTX-M-15_, *bla*_TEM_, *bla*_SHV,_ and *bla*_OXA_), and are resistant to multiple antibiotics of clinical relevance. The presence of ESBL genes in bacteria connects to the co-emergence of resistance against non-beta lactam antibiotics as well. Conclusively, the wild aquatic birds often found in urban and peri-urban areas have the potential to disseminate resistant microflora through their movement and interaction with biotic and abiotic subjects in the environment. The findings of the present study may be helpful for health officials, veterinary professionals and public policymakers to chalk out an effective control and prevention plan by creating public awareness, preserving wildlife, and environmental interventions to slow down the pace of emerging antimicrobial resistance.

## Figures and Tables

**Table 1:** ESBL genes detected in bacteria isolated from different bird species at Trimmu Barrage.

Bird Species	Total ESBL n (%)	ESBL Gene/Gene Combinations Identified						
		*bla*_CTX-M_ n (%)	*bla*_TEM_ n (%)	*bla*_SHV_ n (%)	*bla*_OXA_ n (%)	*bla*_CTX-M_ + *bla*_TEM_ n (%)	*bla*_CTX-M_ + *bla*_SHV_ n (%)	*bla*_TEM_ + *bla*_OXA_ n (%)
Cattle egret (*Bubulcus ibis*)	17 (24.3)	11 (64.7)	3 (17.7)	1 (5.9)	-	2 (11.8)	-	-
Little egret (*Egretta garzetta*)	29 (41.4)	18 (62.1)	5 (17.2)	1 (3.5)	4 (13.8)	-	1 (3.5)	-
Common teal (*Anas crecca*)	20 (28.6)	5 (25)	8 (4)	-	-	5 (25)	-	2 (10)
Chicken (*Gallus gallus domesticus*)	31 (44.3)	16 (51.6)	4 (12.9)	-	2 (6.5)	9 (29)	-	-
Total number	97 (34.6)	50 (51.6)	20 (20.6)	2 (2.1)	6 (6.2)	16 (16.5)	1 (1)	2 (2.1)

**Table 2:** Prevalence of ESBL-producing bacterial species recovered from wild birds and chickens at Trimmu Barrage.

Bird Species	No. of Samples	ESBL Bacterian (%)	*E. coli* n (%)	*E. cloacae* n (%)	*K. pneumoniae* n (%)	*S. enterica* n (%)	*P. aeruginosa* n (%)
Cattle egret *(Bubulcus ibis)*	70	17 (24.3%)	12 (12.37%)	4 (4.12%)	0	1 (1.03%)	0
Little egret *(Egretta garzetta)*	70	29 (41.4%)	24 (24.74%)	1 (1.03%)	2 (2.06%)	1 (1.03%)	1 (1.03%)
Common teal *(Anas crecca)*	70	20 (28.6%)	16 (16.49%)	3 (3.09%)	1 (1.03%)	0	0
Chicken *(Gallus gallus domesticus)*	70	31 (44.3%)	20 (20.62%)	3 (3.09%)	5 (5.15%)	2 (2.06%)	1 (1.03%)
Total number	280	97 (34.6%)	72 (74.2%)	11 (11.3%)	8 (8.3%)	4(4.1%)	2 (2.1%)

**Table 3:** Frequency and diversity of ESBL genetic determinants associated with different bacterial species recovered from wild birds and chickens at Trimmu Barrage.

Bacterial Species	Total ESBL n (%)	ESBL Gene/Gene Combinations Identified						
		*bla*_CTX-M_ n (%)	*bla*_TEM_ n (%)	*bla*_SHV_ n (%)	*bla*_OXA_ n (%)	*bla*_CTX-M_ + *bla*_TEM_ n (%)	*bla*_CTX-M_ + *bla*_SHV_ n (%)	*bla*_TEM_ + *bla*_OXA_ n (%)
*Escherichia coli*	72 (74.2%)	38 (52.8%)	14 (19.5%)	2 (2.8%)	5 (6.9%)	13 (18.1%)	0	0
*Enterobacter cloacae*	11 (11.3%)	6 (54.5%)	4 (36.4%)	0	1 (9.1%)	0	0	0
*Klebsiella pneumoniae*	8 (8.3%)	3 (37.5%)	0	0	0	3 (37.5%)	1 (12.5%)	1 (12.5%)
*Salmonella enterica*	4 (4.1%)	2 (50%)	1 (25%)	0	0	0	0	1 (25%)
*Pseudomonas aeruginosa*	2 (2.1%)	1 (50%)	1 (50%)	0	0	0	0	0
Total number	97 (34.6%)	50 (51.55%)	20 (20.6%)	2 (2.06%)	6 (6.2%)	16 (16.5%)	1 (1.03%)	2 (2.1%)

**Table 4:** Antibiogram of ESBL-producing bacteria isolated from wild birds and chickens at Trimmu Barrage.

Antibiotic	*E. cloacae* (n = 11)			*K. pneumoniae* (n = 8)			*S. enterica* (n = 4)			*P. aeruginosa* (n = 2)			*E. coli* (n = 72)		
	R	S	I	R	S	I	R	S	I	R	S	I	R	S	I
	n (%)														
Streptomycin	1 (9)	10 (90.9)	0	2 (25)	6 (75)	0	2 (50)	2 (50)	0	1 (50)	1 (50)	0	3 (4.16)	69 (95.8)	0
Neomycin	2 (18.2)	8 (72.7)	1 (9)	1 (12.5)	6 (75)	1 (12.5)	1 (25)	3 (75)	0	0	1 (50)	1 (50)	21 (29.2)	46 (63.9)	5 (6.9)
Gentamicin	0	11 (100)	0	3 (37.5)	3 (37.5)	2 (25)	1 (25)	3 (75)	0	0	2 (100)	0	12 (16.7)	55 (76.4)	5 (6.9)
Florfenicol	3 (27.3)	8 (72.7)	0	2 (25)	6 (75)	0	0	4 (100)	0	0	2 (100)	0	7 (9.7)	61 (84.7)	4 (5.6)
Ceftiofur	6 (54.5)	5 (45.5)	0	4 (50)	3 (37.5)	1 (12.5)	2 (50)	2 (50)	0	1 (50)	1 (50)	0	64 (88.9)	8 (11.1)	0
Enrofloxacin	3 (27.3)	6 (54.5)	2 (18.2)	3 (37.5)	5 (62.5)	0	1 (25)	3 (75)	0	0	2 (100)	0	6 (8.3)	65 (90.3)	1 (1.4)
Norfloxacin	4 (36.4)	7 (63.4)	0	2 (25)	6 (75)	0	0	4 (100)	0	0	2 (100)	0	0	70 (97.2)	2 (2.8)
Tylosin	5 (45.5)	5 (45.5)	1 (9)	4 (50)	4 (50)	0	2 (50)	1 (25)	1 (25)	0	1 (50)	1 (50)	11 (15.3)	61 (84.7)	0
Ampicillin	7 (63.6)	4 (36.4)	0	6 (75)	1 (12.5)	1 (12.5)	3 (75)	0	1 (25)	2 (100)	0	0	41 (56.9)	27 (37.5)	4 (5.5)
Doxycycline	3 (27.3)	6 (54.5)	2 (18.2)	1 (12.5)	5 (62.5)	2 (25)	1 (25)	2 (50)	1 (25)	1 (50)	1 (50)	0	14 (19.4)	47 (65.3)	11 (15.3)
Colistin	4 (36.4)	6 (54.5)	1 (9)	0	8 (100)	0	2 (50)	2 (50)	0	0	2 (100)	0	9 (12.5)	60 (83.3)	3 (4.2)
Imipenem	0	11 (100)	0	1 (12.5)	7 (87.5)	0	0	4 (100)	0	0	2 (100)	0	3 (4.2)	69 (95.8)	0

R: Resistant: S: Sensitive; I: Intermediate.

**Table 5:** The overall susceptibility antibiogram of ESBL-producing bacteria isolated from wild birds and chickens at Trimmu Barrage.

Antimicrobials	*E. cloacae*	*K. pneumoniae*	*S. enterica*	*P. aeruginosa*	*E. coli*
Streptomycin	90.9	75	50	50	95.8
Neomycin	72.7	75	75	50	63.9
Gentamicin	100	37.5	75	100	76.4
Florfenicol	72.7	75	100	100	84.7
Ceftiofur	45.5	37.5	50	50	11.1
Enrofloxacin	54.5	62.5	75	100	90.3
Norfloxacin	63.6	75	100	100	97.2
Tylosin	45.5	50	25	50	84.7
Ampicillin	36.4	12.5	0	0	37.5
Doxycycline	54.5	62.5	50	50	65.3
Colistin	54.5	100	50	100	83.3
Imipenem	100	87.5	100	100	95.8
Mean	65.90416667	62.5	62.5	70.83333	73.83833
Standard Deviation	21.62692533	24.42521052	31.07907803	33.4279	26.19029
Standard Error	6.243155581	7.050950935	8.971757032	9.649802	7.560486
Minimum	36.36	12.5	0	0	11.11
Maximum	100	100	100	100	97.22
Count	12	12	12	12	12

**Table 6:** Primers used for detection of ESBL genetic determinants and 16S rRNA gene.

Target Gene	Primers	Primer Sequences (5′-3′)	Annealing Temp. (°C)	Amplicon Size	Reference
*bla*_CTX-M_	F	ATGTGCAGYACCAGTAARGTKATGGC	58	593 bp	[51]
	R	TGGGTRAARTARGTSACCAGAAYCAGCGG			
*bla*_TEM_	F	TCGCCGCATACACTATTCTCAGAATGA	50	445 bp	
	R	ACGCTCACCGGCTCCAGATTTAT			
*bla*_SHV_	F	CGCCGGGTTATTCTTATTTGTCGC	68	1016 bp	[52]
	R	TCTTTCCGATGCCGCCGCCAGTCA			
*bla*_OXA_	F	ATTATCTACAGCAGCGCCAGTG	56	296 bp	[53]
	R	TGCATCCACGTCTTTGGTG			
16S rRNA	fD1	AGAGTTTGATCCTGGCTCAG	52	1500 bp	[54]
	rP2	ACGGCTACCTTGTTACGACTT			

## Data Availability

The data presented in this study are available on request from the corresponding author.
